# Synopsis of Midwifery

**Published:** 1914-03

**Authors:** 


**Synopsis of Midwifery.** By ALECK W. BOURNE, B.A., M.B., B.C. Cantab., F.R.C.S. Pp. 212. Bristol: John Wright & Sons Ltd. 1913. Price 5s. net.

This attempt at preparing a Synopsis of Midwifery is in our opinion a very successful one. The book is very full of detail, but the matter is so condensed as to occupy but little space, and the style though necessarily terse is by no means unpleasant. The arrangement of paragraphs and type admirably fulfils its purpose of presenting the text in a manner easy for reference or committing to memory. To the general description of the mechanism of normal labour we think a brief account of the mechanism in each individual position might have been appended, since this is so frequently an examination question, and the ability to answer it with speed and certainty in accurate, concise language effects great saving of time which may be much needed for the rest of the paper.

We heartily recommend the book to students going up for examinations. It is too full and reasoned to be a mere "cram" book, but will crystallise previously-acquired knowledge into a form easily produced and well adapted to the answering of examination questions.

